# FGFR/RACK1 interacts with MDM2, promotes P53 degradation, and inhibits cell senescence in lung squamous cell carcinoma

**DOI:** 10.20892/j.issn.2095-3941.2020.0389

**Published:** 2021-08-15

**Authors:** Tao Chen, Fei Wang, Shupei Wei, Yingjie Nie, Xiaotao Zheng, Yu Deng, Xubin Zhu, Yuezhen Deng, Nanshan Zhong, Chengzhi Zhou

**Affiliations:** 1 State Key Laboratory of Respiratory Disease, National Clinical Research Center for Respiratory Disease, Guangzhou Institute of Respiratory Health, the First Affiliated Hospital of Guangzhou Medical University, Guangzhou 510120, China; 2 NHC Key Laboratory of Pulmonary Immunological Diseases, Clinical Research Lab Center, Guizhou Provincial People’s Hospital, Guiyang OK 550002, China; 3 Longgang Central Hospital of Shenzhen, Affiliated Shenzhen Longgang Central Hospital of Zunyi Medical College, Shenzhen 518116, China; 4 Center for Molecular Medicine, Xiangya Hospital, Central South University, Changsha 410078, China

**Keywords:** FGFR, RACK1, MDM2, P53, senescence

## Abstract

**Objective:**

FGFR is considered an important driver gene of lung squamous cell carcinoma (LSCC). Thus, identification of the biological events downstream of FGFR is important for the treatment of this malignancy. Our previous study has shown that the FGFR/RACK1 complex interacts with PKM2 and consequently promotes glycolysis in LSCC cells. However, the biological functions of the FGFR/RACK1 complex remain poorly understood.

**Methods:**

Anchorage-independent assays and *in vivo* tumorigenesis assays were performed to evaluate cancer cell malignancy. Distant seeding assays were performed to evaluate cancer cell metastasis. β-gal staining was used to examine cell senescence, and immunoprecipitation assays were performed to examine the interactions among FGFR, RACK1, and MDM2.

**Results:**

FGFR/RACK1 was found to regulate the senescence of LSCC cells. Treatment with PD166866, an inhibitor of FGFR, or knockdown of RACK1 induced senescence in LSCC cells (*P* < 0.01). A molecular mechanistic study showed that FGFR/RACK1/MDM2 form a complex that promotes the degradation of p53 and thus inhibits cell senescence. PD166866 and RG7112, an MDM2/p53 inhibitor, cooperatively inhibited the colony formation and distal seeding of LSCC cells (*P* < 0.01), and upregulated the expression of p53 and p21.

**Conclusions:**

Together, our findings revealed the regulatory roles and mechanisms of FGFR/RACK1 in cell senescence. This understanding should be important in the treatment of LSCC.

## Introduction

Lung cancer is one of the most malignant tumors wordwide^1,2^ and is the malignancy with the highest morbidity and mortality in China^3^. The FGF/FGFR tyrosine kinase signaling pathway, which regulates various biological events, including embryonic development and tissue repair^4^, plays an important role during tumorigenesis and can lead to chemotherapeutic resistance if it is overactivated^5^.

The FGFR family includes 4 members: FGFR1, FGFR2, FGFR 3, and FGFR4^6^. Each receptor contains an extracellular domain, a transmembrane domain, and an intracellular domain. The FGF ligands comprise 23 members, whose binding to FGFR mediates downstream signal transduction events^7^. FGFR abnormalities are very common in squamous non-small cell lung cancer^8–10^. Amplification of the FGFR gene can be detected in approximately 20% of lung squamous cell carcinomas^11^. Therefore, elucidation of the biological functions of FGFR and the downstream biological events is urgently needed for the treatment of lung squamous cell carcinomas. Several inhibitors targeting FGFR1 (such as PD166866) have entered clinical trials^12^.

RACK1 (the receptor for activated PKC kinase) is a 7 WD40-repeat-containing adaptor protein that acts as an integrated hub for different signaling pathways in functions including cell growth, migration, and tumorigenesis^13^. Recent studies have reported that RACK1 has very important roles in in lung cancer^14–16^, through promoting the cell growth and migration of lung cancer cells by activating Hedgehog signaling, although the exact mechanism requires further exploration^15^.

Extensive evidence indicates that senescence is a mechanism that suppresses tumourigenesis^17^. Senescence is activated by stress^18^. Cell cycle arrest may occur in the division and proliferation of cells through senescence, and the cellular morphology also changes significantly. Senescent cells are positive for β-galactosidase staining, and cell cycle arrest-related signaling pathways, such as P53-P21 and P16-Rb, become activated^19^. Chemotherapy, radiotherapy, and hypoxia are inducers of cellular senescence^20^.

We previously found that the adaptor protein RACK1 interacts with FGFR and consequently promotes the phosphorylation of PKM2 by FGFR^16^, thereby increasing tumor cell glycolysis. However, whether RACK1 mediates other downstream biological functions of FGFR remains unclear. In this article, we investigated the effect of the FGFR/RACK1 complex on cellular senescence in lung squamous cell carcinomas and determined the underlying mechanism.

## Materials and methods

### Cell culture

The lung cancer cell lines NCI-H226 and H1703 were purchased from the Shanghai Cell Bank of the Chinese Academy of Sciences. Cells were cultured with DMEM containing 10% serum and penicillin-streptomycin solution. Cells were placed in an incubator at 37° and 5% CO_2_. Cell transfection was mainly performed with Lipofectamine 2000 and was conducted according to the manufacturer’s instructions. Forty-eight hours after cell transfection, 1 µg/mL of puromycin was used for screening. Seven days later, the surviving cells were mixed, and gene expression was detected with Western blot analyses.

### Western blot analysis

After the cells were washed twice with PBS, they were lysed on ice for 15 min with RIPA buffer (1% NP-40, 0.1% SDS, 0.5% deoxycholate, 50 mM Tris (pH 7.4), and protease inhibitor cocktail) and collected with a scraper. After centrifugation at 12,000 rpm at 4 °C for 15 min, the protein concentration was determined with Bradford reagent (Sigma). The protein samples were mixed with 6× loading buffer and boiled for 5 min, and electrophoresis was then performed. The proteins were subsequently transferred to polyvinylidene difluoride membranes (Millipore). The membranes were incubated with TBST containing 5% skimmed milk (25 mM Tris, 150 mM NaCl, and 0.05% Tween 20, pH 7.5) for 1 h at room temperature and then incubated overnight at 4 °C with primary antibody. After the samples were washed 3 times with TBST, horseradish peroxidase-conjugated secondary antibody was added. The cells were then incubated for 1 h at room temperature, washed 3 times with TBST, and then developed with an enhanced chemiluminescence system (Pierce).

### Soft agar assays

Soft agar assays were performed in 12-well plates. The soft agar contained an upper and lower layer. The lower layer of agar, which was used to embed the 12-well plate, had an agar concentration of 0.5% and a serum concentration of 10%. The upper layer had an agar concentration of 0.35% and a serum concentration of 10% for cell resuspension. An inhibitor (PD166866 or RG7112) was mixed in the upper layer. After the agar was heated to 37 °C, the agar in the lower layer was poured. A total of 2,000 cells were added to the upper layer and seeded on the lower layer after being mixed well. The cells were cultured at 37 °C for 14 days and then photographed and counted.

### Tumorigenesis

Four-week-old male nude mice were divided into 2 groups with 4 mice per group. One group was injected with cancer cells with the shRNA control (1 × 10^6^ cells/point), and the other group was injected with cancer cells with shRACK1 (1 × 10^6^ cells/point). The mice were euthanized 5 weeks later, the tumor tissues were removed and weighed, and RACK1 expression was detected through immunohistochemistry. The experiments and the related analysis were approved by the Laboratory Animal Ethics Committee of the First Affiliated Hospital of Guangzhou Medical University (approval No. 2019-099). All applicable international, national, and/or institutional guidelines for the care and use of animals were followed.

### Immunohistochemistry

Paraffin sections were dewaxed and hydrated with xylene and gradient ethanol, after which antigen repair was performed with sodium citrate solution. The endogenous peroxidase activity was activated with 0.3% hydrogen peroxide, and the sections were blocked with 5% BSA. The sections were then incubated with primary antibody overnight at 4 °C with the ZSGB-BIO immunohistochemistry kits. On the following day, the sections was washed 3 times with PBST, incubated with secondary antibody for 2 h at room temperature and then developed for 2 min with DAB. After development, staining was performed with hematoxylin to visualize the nuclei. The sections were treated with 1% hydrochloric acid/ethanol solution for 6 s and washed.

### GST pull-down

Cells were collected, lysed, and centrifuged at 4 °C and 12,000 rpm for 20 min to obtain the supernatant, which was then incubated with 10 µg of GST and GST-RACK1 fusion protein at 4 °C overnight. Sepharose 4B GST gel beads were then added and incubated for an additional 4 h. The gel beads were washed 3 times with PBST buffer for 5 min each time; 30 µL of loading buffer was then added and boiled for 5 min at 100 °C. The supernatant was used for Western blot analysis.

### Co-immunoprecipitation

NCI-H226 and H1703 cells were collected, lysed, and centrifuged at 4 °C and 12,000 rpm for 20 min to obtain the supernatant, which was then mixed with primary antibody and incubated overnight at 4 °C. Protein A beads were then added and incubated for an additional 4 h. The beads were washed 3 times with PBST buffer for 5 min each time; 30 µL of loading buffer was then added and boiled for 5 min at 100 °C. The supernatant was used for Western blot analysis.

### Senescence analysis

Cultured cells were plated in 6-well dishes 1 day before staining. Cells were fixed and stained according to the manufacturer’s protocol (Senescence β-Galactosidase Staining Kit, Cell Signaling Technology #9860). Briefly, cells were fixed in a 2% formaldehyde and 0.2% glutaraldehyde solution for 15 min, washed in PBS, and stained overnight at 37 °C in an X-gal staining solution. Stained cells were imaged with inverted microscopy. Ten random images were collected, and cells were counted.

### Ubiquitination assays

The lysate was first prepared by suspension of cells in IP lysis buffer for 30 min at 4 °C. Immunoprecipitation was performed with 1 mg of protein and 1 µg of primary antibody in 500 µL of IP lysis buffer at 4 °C overnight. The reaction mixtures were incubated with Protein A and Protein G Magnetic Beads (50 µL) at 4 °C for 1 h on a rotator. The immunoprecipitated complexes were washed 3 times with IP wash buffer. The washed beads were incubated with 5× reducing loading buffer and boiled at 100 °C for 5 min. The proteins released from components of the complexes were examined by SDS-polyacrylamide gel electrophoresis and Western blot with anti-ubiquitin antibodies (Cell Signaling Technology, #3933).

### Distal seeding assays

Six-week-old male nude mice were injected with 1 × 10^6^ cells/animal. Two days later, the intervention was performed with inhibitors. Eight weeks later, the mice were sacrificed, lung tissue samples were collected, and the number of lung metastases was detected through HE staining. All animal experiments were approved by the GMU Biomedical Ethics Committee.

## Results

### The FGFR inhibitor PD166866 induces senescence in lung squamous cell carcinoma cells

**Figure 1:**
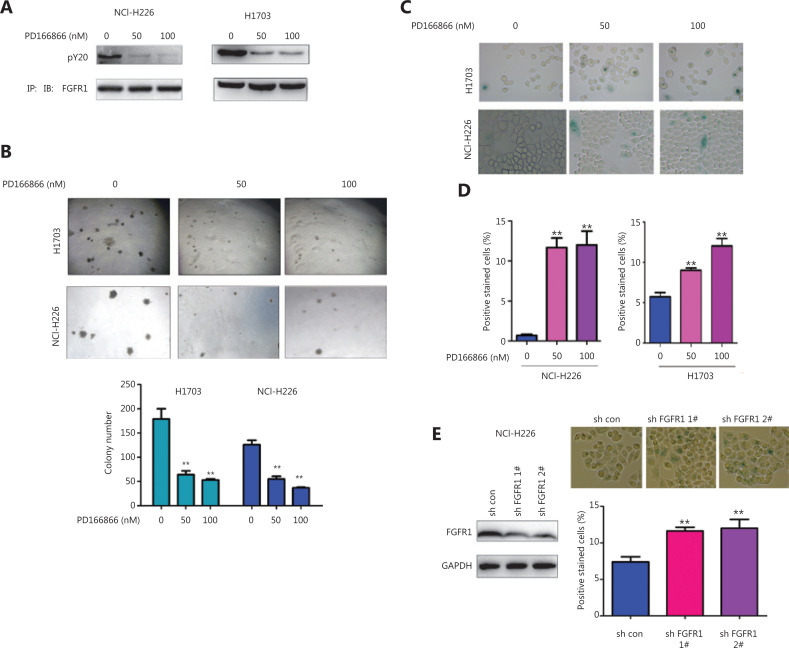
PD166866 induces senescence of lung squamous cell carcinoma cells. (A) NCI-H226 and H1703 cells were treated with PD166866, and the tyrosine phosphorylation of FGFR was detected with immunoprecipitation and Western blot analyses. (B) NCI-H226 and H1703 cells were treated with PD166866, and anchorage-independent growth was detected with soft agar colony assays. (C, D) NCI-H226 and H1703 cells were treated with PD166866, and β-gal staining was used to detect cell senescence. Statistical analyses were performed. (E) FGFR 1 was knocked down in NCI-H226 cells, and β-gal staining was used to detect cell senescence. Statistical analyses were performed. **P < 0.01.

To reveal the function of FGFR in lung squamous cell carcinomas, we first treated the lung squamous cell carcinoma cell lines NCI-H226 and H1703 with the FGFR inhibitor PD166866 and then detected the effect of PD166866 treatment on the phenotypes of the NCI-H226 and H1703 cells. The PD166866 treatment significantly inhibited the FGFR tyrosine phosphorylation level (an important marker of FGFR activation) (**Figure 1A**). In addition, PD166866 treatment inhibited the colony formation of the NCI-H226 and H1703 cells on soft agar (**Figure 1B**). Further studies revealed that PD166866 treatment promoted the senescence of the NCI-H226 and H1703 cells (**Figure 1C–1D**). Consistently, knockdown of FGFR1 induced cell senescence in NCI-H226 cells (**Figure 1E**). These observations indicated that the inhibition of FGFR activity caused the senescence of lung squamous cell carcinoma.

### RACK1 knockdown induces senescence in lung squamous cell carcinoma cells

**Figure 2:**
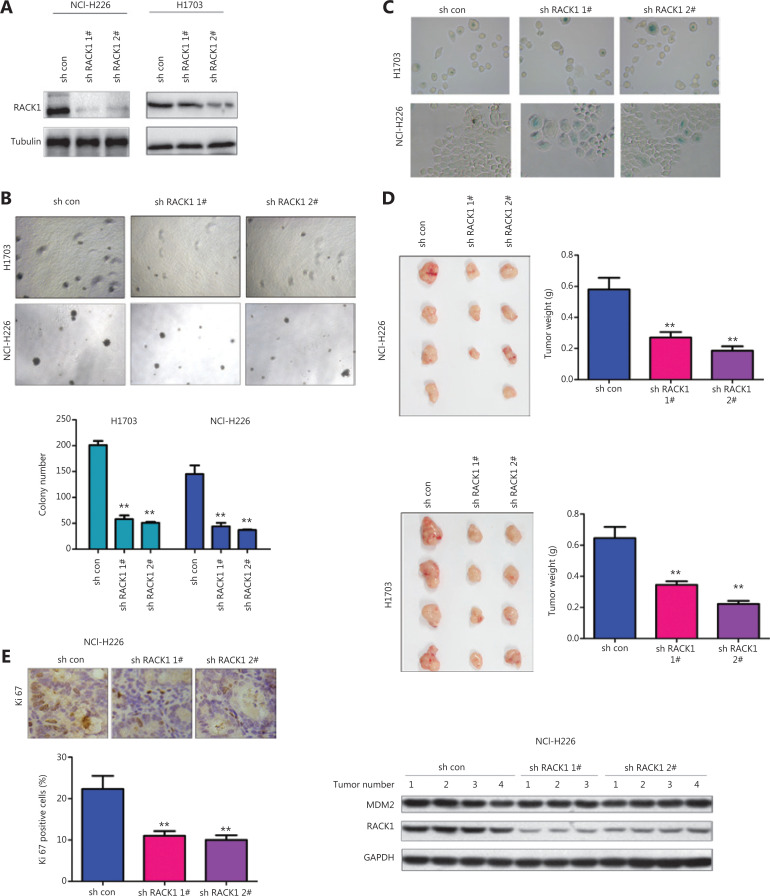
Knockdown of RACK1 induces senescence of lung squamous cell carcinoma cells. (A) RACK1 expression in NCI-H226 and H1703 cells was detected. (B) RACK1 was knocked down in NCI-H226 and H1703 cells, and anchorage-independent growth was detected with colony formation assays. (C) β-gal staining was used to detect cell senescence. (D) RACK1 expression was knocked down in NCI-H226 and H1703 cells, subcutaneous tumorigenesis assays were performed, and the tumor weights were statistically analyzed. (E) Immunohistochemistry was used to detect the expression of Ki67 in tumors derived from RACK1 knockdown NCI-H226 cells (20x). Left: representative immunohistochemistry image; right: RACK1 and MDM2 protein levels in tumors. **P < 0.01.

In our previous studies, we have found that FGFR interacts with RACK1^16^. Therefore, we studied the functions of RACK1 expression in FGFR-mediated cellular senescence. First, we knocked down the expression of RACK1 in NCI-H226 and H1703 cells (**Figure 2A**), and detected RACK1 function with soft agar assays (**Figure 2B**). RACK1 knockdown inhibited the formation of colonies of NCI-H226 and H1703 cells in soft agar. In agreement with the results of PD166866 treatment, RACK1 knockdown promoted the senescence of NCI-H226 and H1703 cells (**Figure 2C**). In the *in vivo* experiments, RACK1 knockdown inhibited the *in vivo* tumorigenesis of NCI-H226 and H1703 cells, as shown by the tumor size and tumor weight (**Figure 2D**). Moreover, lower intensity Ki67 staining was observed in the tumors derived from cells with knockdown of RACK1 (**Figure 2E**).

### FGFR forms a complex with RACK1 and MDM2 in lung squamous cell carcinoma

**Figure 3:**
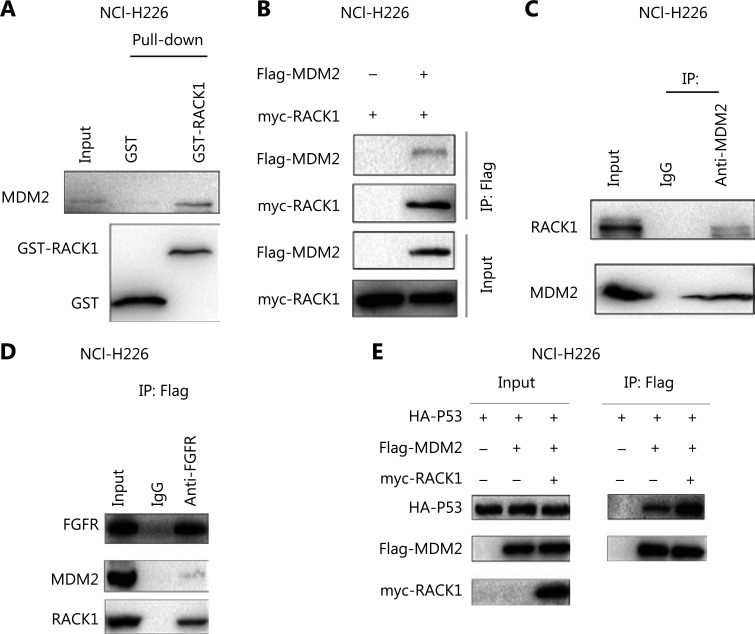
Interaction between RACK1 and MDM2. (A) The interaction between the GST-RACK1 fusion protein and MDM2 was detected with GST pull-down assays. Ten micrograms of the fusion protein GST-RACK1 was incubated with NCI-H226 cell lysate. (B) The interaction between myc-RACK1 and Flag-MDM2 expressed in NCI-H226 cells was detected by immunoprecipitation. Four micrograms of myc-RACK1 and Flag-MDM2 plasmids was transfected into the cells, and the proteins were collected for immunoprecipitation 48 h later. (C) The interaction between RACK1 and MDM2 expressed in NCI-H226 cells was detected by immunoprecipitation. (D) The interactions among FGFR, RACK1, and MDM2 expressed in NCI-H226 cells were detected by immunoprecipitation. (E) The interaction between the exogeneous proteins myc-RACK1 and Flag-MDM2 expressed in NCI-H226 cells was detected by immunoprecipitation. Four micrograms of myc-RACK1 and Flag-MDM2, HA-p53, and Flag MDM2 plasmids was transfected into the cells, which were collected 48 h after transfection.

The MDM2-P53 signaling pathway has an important role in cellular senescence. We detected the interaction between FGFR/RACK1 and important components of the MDM2-P53 signaling pathway. In GST pull-down experiments, the fusion proteins GST-RACK1 and MDM2 formed a complex (**Figure 3A**). In coimmunoprecipitation experiments, we observed an interaction between exogenously expressed RACK1 (myc-RACK1) and MDM2 (Flag-MDM2) (**Figure 3B**). Moreover, the endogenously expressed RACK1 interacted with MDM2 (**Figure 3C**). After coimmunoprecipitation with an antibody to FGFR, both RACK1 and MDM2 were detected (**Figure 3D**), thus indicating that FGFR/RACK1/MDM2 had formed a ternary complex. Functionally, RACK1 promoted the interactions between P53 and MDM2 (**Figure 3E**).

### Inhibition of P53 protein levels by the FGFR/RACK1 complex

**Figure 4:**
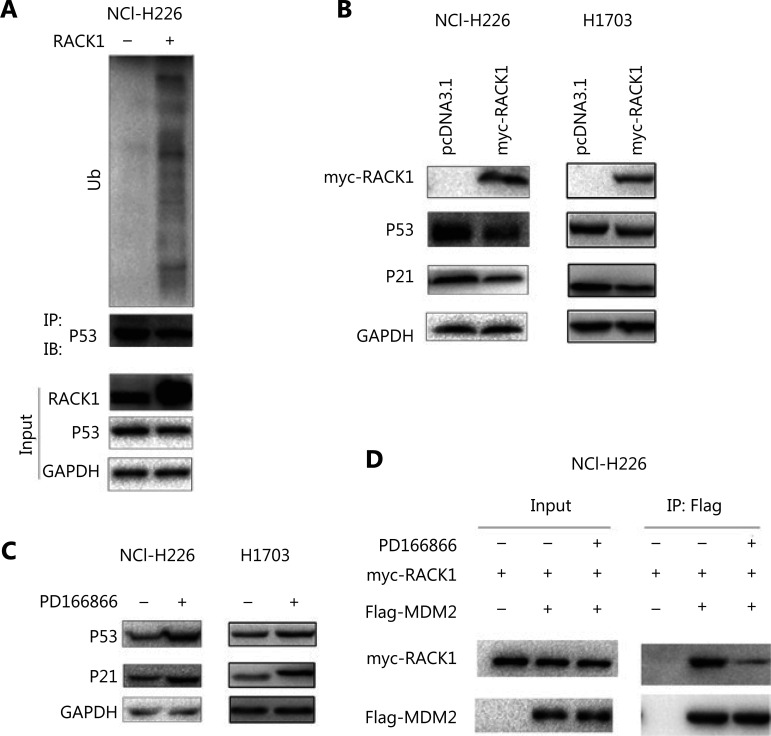
RACK1 promotes the ubiquitination of p53. (A) Overexpression of RACK1 in NCI-H226 cells promoted the ubiquitination of p53. (B) Overexpression of RACK1 in NCI-H226 and H1703 cells inhibited the p53 and p21 protein levels. (C) Treatment of NCI-H226 and H1703 cells with PD166866 increased the protein levels of p53 and p21. (D) The effects of PD166866 on the interaction between RACK1 and MDM2 were detected by immunoprecipitation.

We investigated the effect of the FGFR/RACK1 complex on P53 protein levels. In NCI-H226 cells, the upregulation of RACK1 expression promoted the degradation of P53 through the ubiquitination pathway (**Figure 4A**). In NCI-H226 and H1703 cells, the protein levels of P53 and P21 were downregulated by overexpression of RACK1 (**Figure 4B**). NCI-H226 and H1703 cells treated with PD166866 showed upregulated P53 and P21 protein levels (**Figure 4C**). In addition, PD166866 treatment inhibited the interaction between RACK1 and MDM2 (**Figure 4D**). These results indicated that the FGFR/RACK1/MDM2 complex inhibits P53 protein levels by promoting P53 ubiquitination.

### Treatment with FGFR and P53-MDM2 inhibitors suppresses lung cancer progression

**Figure 5:**
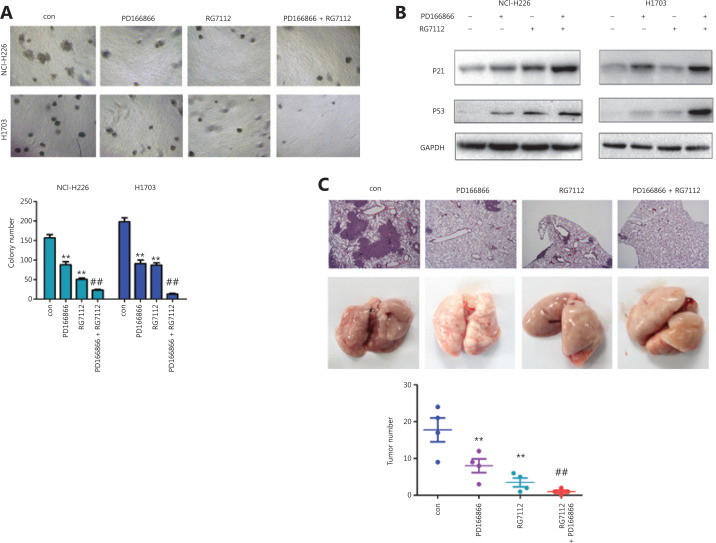
PD166866 and RG7112 cooperatively inhibit the malignancy of lung cancer. (A) PD166866 combined with RG7112 inhibited the colony formation of NCI-H226 and H1703 cells. (B) PD166866 combined with RG7112 promoted the expression of p53 and p21. (C) PD166866 combined with RG7112 inhibited the distal seeding of NCI-H226 cells. The gross morphology of lungs is shown. H&E staining was performed, and the numbers of foci were analyzed (10x). **P < 0.01; ^##^*P* < 0.01.

We investigated the therapeutic effects of combining FGFR and P53-MDM2 inhibitors in lung cancer treatment. The FGFR inhibitor PD166866 and the P53-MDM2 inhibitor RG7112 cooperatively inhibited the colony formation of NCI-H226 and H1703 cells (**Figure 5A**). Consistently, PD166866 and RG7112 jointly induced the expression of P53 and P21 proteins (**Figure 5B**). In model mice, PD166866 and RG7112 together inhibited the distal implantation ability of NCI-H226 cells (**Figure 5C**).

## Discussion

FGFR amplification is very common in lung squamous cell carcinoma. FGFR is considered an important driver gene in lung squamous cell carcinoma^8,9,21^. Moreover, FGFR amplification is also found in cholangiocarcinoma^22^. Currently, the treatment strategies for lung squamous cell carcinoma and are limited. Therefore, elucidation of the biological events mediated by the FGFR pathway is important. In this study, we found that FGFR inhibitors induced cell senescence by increasing P53 protein levels, thereby inhibiting the formation of lung carcinoma cell colonies in soft agar and their distal implantation in mice. These findings expand understanding of the downstream biological functions of FGFR.

**Figure 6:**
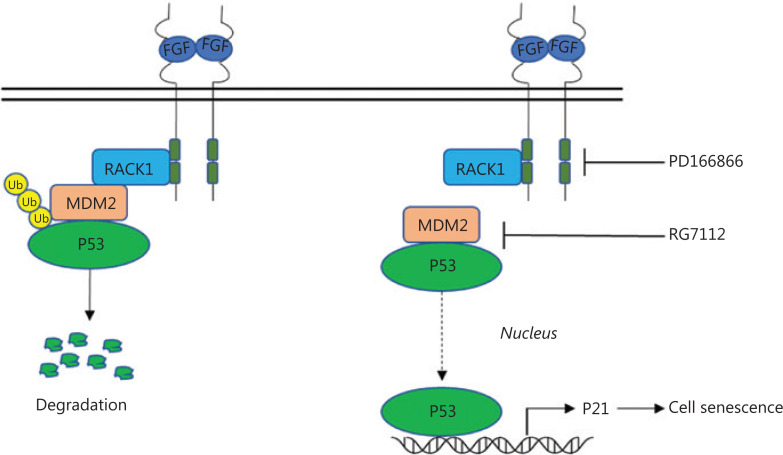
Schematic of the proposed mechanism(s) for FGFR/RACK1 interaction with MDM2 to inhibit the senescence of lung squamous cell carcinoma. The interaction of FGFR/RACK1 with MDM2 promotes the degradation of P53 (left); the FGFR inhibitor PD166866 and the MDM2/p53 inhibitor RG7112 cooperatively decrease the interactions between RACK1 and MDM2; weaken the interaction between MDM2/p53; and induce the accumulation of p53 and the upregulation of p21 and consequently cell senescence.

Notably, we found that the RACK1/MDM2 complex regulates the stability of the P53 protein. In previous studies, we have found that FGFR and RACK1 forms a complex that promotes the phosphorylation of PKM2^16^. In this study, the FGFR/RACK1 complex was found to interact with MDM2, thereby enhancing the interaction between MDM2 and P53, and inhibiting the P53 protein levels. These observations suggested that the complex of FGFR/RACK1 exerts multiple functions through interacting with different effectors. The FGFR inhibitor PD166866 inhibited the phosphorylation of FGFR1, thus decreasing the interaction between RACK1 and MDM2 and weakening the interactions among RACK1, MDM2 and P53. The accumulation of P53 protein activated P21 expression and induced cellular senescence (**Figure 6**). These experimental results indicated that the FGFR tyrosine kinase functions as a regulator of senescence.

Multiple signaling pathways are mediated by FGFR^23^. Another interesting result in this study was the therapeutic effect resulting from combining an FGFR inhibitor with an MDM2-P53 inhibitor. Together, these inhibitors synergistically induced cellular senescence and inhibited the distal seeding of lung squamous cell carcinoma cells. Our results suggest that this combination of inhibitors may be an effective method for treating lung cancer with FGFR amplification in clinical trials.

## Conclusions

In this study, we uncovered the effects and related mechanisms underlying the induction of senescence in lung squamous cell carcinoma by FGFR inhibitors, thus potentially providing a new strategy for the treatment of lung squamous cell carcinoma.
